# Genome Sequence and Attenuating Mutations in West Nile Virus Isolate from Mexico

**DOI:** 10.3201/eid1012.040647

**Published:** 2004-12

**Authors:** David W.C. Beasley, C. Todd Davis, Jose Estrada-Franco, Roberto Navarro-Lopez, Arturo Campomanes-Cortes, Robert B. Tesh, Scott C. Weaver, Alan D.T. Barrett

**Affiliations:** * University of Texas Medical Branch, Galveston, Texas, USA;; † Comision Mexico-Estados Unidos para la Prevencion de la Fiebre Aftosa y Otras Enfermedades Exoticas de los Animales, Mexico City, Mexico

**Keywords:** West Nile virus, arbovirus, flavivirus, Mexico, genome sequence, dispatch

## Abstract

The complete genome sequence of a Mexican West Nile virus isolate, TM171-03, included 46 nucleotide (0.42%) and 4 amino acid (0.11%) differences from the NY99 prototype. Mouse virulence differences between plaque-purified variants of TM171-03 with mutations at the E protein glycosylation motif suggest the emergence of an attenuating mutation.

Since its introduction into North America in 1999, West Nile virus (WNV) has spread rapidly across the continent, and evidence for virus circulation has also been detected in the Caribbean and parts of Central America (*1*). In 2003, WNV was isolated from a dead raven in Villahermosa, in the state of Tabasco, Mexico (*2*). Nucleotide sequencing of the premembrane (prM) and envelope (E) structural protein genes of this strain, TM171-03, and comparison with sequences from other North American isolates indicated that this virus had accumulated several unique mutations from the New York 1999 strain 382-99 (NY99) prototype sequence. We describe the complete genomic sequence of TM171-03 and its relationship to other North American isolates, as well as the results of virulence phenotype comparisons.

## The Study

The isolation and initial characterization of TM171-03 have been described elsewhere (*2*). For genomic sequencing, RNA was extracted from infected Vero cell culture supernatant (second Vero cell passage from the original brain material, designated V2) using the QiaAmp kit (Qiagen Inc., Valencia, CA), reverse transcribed with AMV reverse transcriptase (RT) (Roche, Indianapolis, IN) and amplified by polymerase chain reaction (PCR) as nine overlapping fragments by using Taq polymerase (Roche). The PCR products were purified from 1.5% TAE/agarose gels by using the QiaQuick kit (Qiagen) and directly sequenced on an ABI Prism model 3100 DNA sequencer (Applied Biosystems, Foster City, CA) at the University of Texas Medical Branch's Protein Chemistry Core Facility by using the amplifying primers and additional internal primers. The primers used for RT-PCR and sequencing were similar to those used by Lanciotti et al. (*3*) and Beasley et al. (*4*) (complete details are available on request). Sequence data were assembled into the complete genome sequence and analyzed as described elsewhere (*2**,**4*). In addition, Bayesian analyses were performed by using MRBAYES v3.0 (*5*) and 100,000 generations. A general time-reversible model was used with empirically estimated base frequencies and either a codon position-specific or a γ distribution of substitution rates.

**Figure 1:**
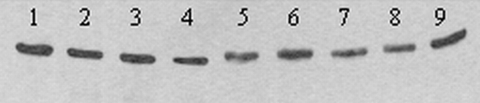
Western blot showing differing mobility of E proteins from nine plaque-purified variants of West Nile virus (WNV) strain TM-171 Mex03. Nucleotide sequencing of strains in lanes 5 to 9 indicated the presence of an "NYS" glycosylation motif at residues 154 to 156 of E, while strains in lanes 1 to 4 encoded "NYP." Antigens were separated in a nonreducing 5%/10% discontinuous sodium dodecyl sulfate–polyacrylamide gel, transferred to 0.2 μm nitrocellulose and detected with WNV-specific monoclonal antibody 7H2 (*6*).

The genomic sequence for TM171-03 (GenBank accession number AY660002) differed from the NY99 prototype sequence (GenBank AF196835) at 46 nucleotides (nt) (0.42%). As reported previously, sequencing the prM-E genes of TM171-03 (V1 passage) identified nonsynonymous mutations encoding substitutions at prM-141 Ile→Thr and E156 Ser→Pro (*2*). The E-156 mutation results in the loss of the E-154-156 "NYS" glycosylation motif. Complete genome sequencing identified only two other encoded amino acid changes from the NY99 sequence at NS4B-245 (Ile→Val) and NS5-898 (Thr→Ile). However, during the sequencing of the V2 passage material, a reversion from Pro to Ser encoded at E-156 was observed. Analysis of the sequence chromatograms from V1 and V2 passages, and for a PCR product obtained from the original brain material, indicated that this reversion was likely to be the result of a mixed virus population, with overlapping "T" and "C" peaks visible at residue 1432 in the sense or antisense sequences for each product. To confirm this finding, PCR products containing the E-156 region from each passage level of TM171-03 were cloned into pGEM-T(Easy) (Promega, Madison, WI), and five clones were sequenced for each. For the original brain tissue, four clones encoded Pro at E-156 and one clone encoded Ser. For products derived from either V1 or V2 passages, two or three clones encoded a Pro at E-156, and the remainder encoded a Ser. In addition, several variants of TM171-03 were purified through two rounds of plaque selection in Vero cells, and nucleotide sequencing of these variants also confirmed a mixed population. Sequences from approximately half of the plaques encoded a Pro at E-156, while the remainder encoded Ser. Western blotting of infected Vero cell lysate antigens for these variants with WNV E protein-specific monoclonal antibody 7H2 (*6*) showed differences in the electrophoretic mobility of the proteins consistent with the presence or absence of glycosylation (Figure 1).

**Figure 2:**
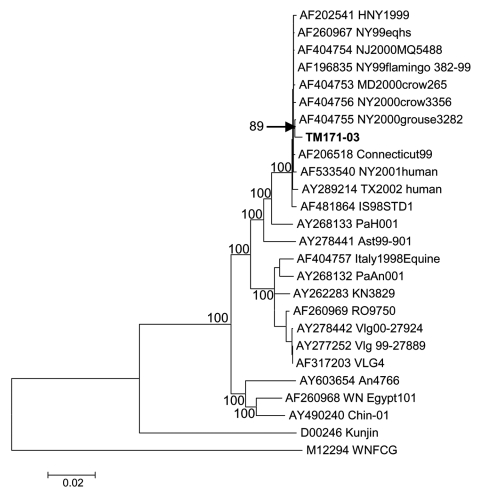
Neighbor-joining phylogenetic tree based on complete genome sequences of West Nile virus strains. Strain TM171-03 is indicated in bold text. The topology of maximum parsimony and maximum likelihood trees was essentially identical. Bayesian analysis also confirmed the close relationship between TM171-03 and NY00-grouse3282 sequences (data not shown). Bootstrap values are shown for major branches (500 replicates). GenBank accession numbers for sequences used to construct the tree are indicated on the branches.

Comparison of the TM171-03 nucleotide sequence with other genomic sequences of WNV strains showed that it was most closely related to strain NY00-grouse3282 (GenBank AF404755; Figure 2). The NY00-grouse3282 sequence differed from NY99 at only 13 nt (0.11%), and its relationship to TM171-03 was apparently based on 10 nonstructural protein region nucleotide differences from NY99 that were shared with TM171-03 (Table 1). None of these mutations encoded amino acid differences, and TM171-03 differed from NY00-grouse3282 at 39 other nucleotides. These were primarily additional mutations that had accumulated in the TM171-03 strain. Genomic sequence data from other East Coast U.S. isolates collected during 2000 and subsequent years are needed to attempt to establish a definitive relationship for TM171-03 with a particular North American isolate.

**Table 1:** Summary of nucleotide and amino acid differences between West Nile virus strains NY99 (AF196835), NY00-grouse3282 (AF404755), and TM171-03

Nucleotide (amino acid)	NY99	NY00	TM171-03
50	A	A	G
71	A	A	G
93	C	T	C
381	C	C	T
483	C	C	T
858	C	C	T
887 (prM-141)	T (Ile)	T (Ile)	C (Thr)
1137	C	C	T
1285	C	T	C
1432 (E-156)^b^	T (Ser)	T (Ser)	C (Pro)
1626	C	C	T
2328	C	C	T
2388	C	C	T
2466	C	C	T
2607	T	T	C
2832	T	T	C
2865	C	C	T
3111	G	G	A
**4146**	**A**	**G**	**G**
4212	T	T	A
**4564**	**T**	**C**	**C**
4749	C	C	T
6120	C	C	T
**6138**	**C**	**T**	**T**
6141	C	C	T
6279	G	G	A
**6426**	**C**	**T**	**T**
6495	G	G	A
**6771**	**A**	**G**	**G**
**7015**	**T**	**C**	**C**
7359	C	C	T
7648 (NS4B-245)	A (Ile)	A (Ile)	G (Val)
7672	C	C	T
**7938**	**T**	**C**	**C**
8067	A	G	A
8109	C	C	T
8676	A	A	G
**8811**	**T**	**C**	**C**
8838	T	T	C
8994	T	T	C
9378	T	T	C
9408	T	T	C
9453	C	C	T
10317	C	C	T
10373 (NS5-898)	C (Thr)	C (Thr)	T (Ile)
**10393**	**C**	**T**	**T**
10828	T	T	G
**10851**	**A**	**G**	**G**
10989	G	G	A

^a^**Bold text** indicates residues at which NY00-grouse 3282 and TM171-03 both differed from NY99. ^b^Strain TM171-03 had a mixed sequence at this residue. Consensus sequence of early passage material had "C" at nucleotide 1432.

To assess the effects of the E-156 Ser→Pro mutation on the virulence of TM171-03, serial 10-fold doses from 1,000 to 0.1 PFU of TM171-03 and four plaque-purified (pp) substrains (TM171-03-pp1 and -pp2 encoding Pro at E-156; TM171-03-pp5 and -pp6 encoding Ser) were inoculated intraperitoneally (i.p.) and intracranially (i.c.) into groups of 3- to 4-week-old female NIH Swiss mice to determine mouse neuroinvasiveness and neurovirulence, as described elsewhere (*7*) and in accordance with guidelines of the University of Texas Medical Branch Institutional Animal Care and Use Committee.

TM171-03 was highly virulent following i.p. and i.c. inoculation, as were the plaque-purified variants, TM171-03-pp5 and -pp6, which encoded the E154-156 NYS glycosylation motif (i.p. and i.c. 50% lethal dose [LD_50_] values for each <2.0 PFU; Table 2). The lethality of these strains was comparable to that of other North American isolates that have been evaluated by using the NIH Swiss mouse model (*4**,**7*). In contrast, the nonglycosylated variants TM171-03-pp1 and -pp2 were both attenuated, having i.p. LD_50_ values >1,000 PFU and i.c. LD_50_ values of 32 and 25 PFU, respectively.

**Table 2:** Neuroinvasiveness and neurovirulence of TM-171 Mex03 parental isolate and plaque-purified variants after injection into 3- to 4-week-old female NIH Swiss mice^a^

Virus	E154-156 sequence	i.p. LD_50_ (PFU)	i.p. AST ± SD (d)^b^	i.c. LD_50_ (PFU)	i.c. AST ± SD (d)^b^
TM171-03	NYP/S	1.3	7.9 ± 0.7	0.8	6.2 ± 1.8
TM171-03-pp1	NYP	>1000	NA	32	6.0 ± 0.9
TM171-03-pp2	NYP	>1000	NA	25	6.7 ± 1.7
TM171-03-pp5	NYS	2.0	9.0 ± 1.4	2.0	7.4 ± 1.2
TM171-03-pp6	NYS	2.0	8.5 ± 1.7	1.3	7.4 ± 0.9

^a^NIH, National Institutes of Health; AST, average survival time; i.p., intraperitoneal; i.c., intracranial; LD_50_, 50% lethal dose; SD, standard deviation; NA, not applicable. ^b^Average survival time ± SD was calculated for

To confirm that the mouse virulence differences between the plaque-purified variants could be primarily attributed to the mutation at E-156, regions that encoded the additional consensus amino acid mutations at prM-141, NS4B-245, and NS5-898 were sequenced. All four plaque-purified variants encoded the three amino acid mutations that were present in the consensus sequence. No additional mutations encoding amino acid changes were identified in the regions that were sequenced for these strains (equivalent to ≈3,000 nt in total for each). Although the entire genome of each plaque-purified variant was not sequenced, we believe that it is highly unlikely that the mouse virulence differences observed between the variants would be attributable to other amino acid mutations in the unsequenced regions that were present in the two E-156 Pro variants but not the E-156 Ser variants or the parental TM171-03 strain.

## Conclusions

These results are somewhat contrary to previously reported data that described attenuated variants with glycosylated E proteins that were derived from a virulent, nonglycosylated Israeli lineage 1 WNV strain (*8*). However, subsequent studies identified E glycosylated variants of the same strain that retained a virulent phenotype, which suggests that multiple determinants, most probably including mutations in the nonstructural protein genes, were responsible for the observed variations in virulence (*9*). Other comparisons of wild-type WNV strains suggested that absence of E protein glycosylation might be associated with attenuation of mouse neuroinvasiveness (*7*). Recently, some of us have shown that mutating the E protein gene of a WNV infectious clone derived from the NY99 prototype strain to prevent glycosylation resulted in a ≈200-fold attenuation of neuroinvasiveness, but not neurovirulence, in the NIH Swiss mouse model (D.W.C. Beasley, et al., unpub. data). Given the greater degree of attenuation of neuroinvasiveness and neurovirulence observed for the nonglycosylated TM171-03-pp1 and -pp2 variants described here, we hypothesize that one or more of the other mutations (at prM-141, NS4B-245, or NS5-898) also contributed to the phenotype, but this hypothesis remains to be determined experimentally. All of these mutations in the absence of the E-156 Ser→Pro mutation (as occurred in the TM171-03-pp4 and -pp5 variants) did not appear to significantly affect the mouse virulence phenotype.

E protein glycosylation appears to play an important role in flavivirus assembly in mammalian cell culture (*10*); the mechanism by which this particular mutation would emerge in a wild-type WNV population, as is the case with the TM171-03 isolate, is not clear. However, the posttranslational processing of glycoproteins differs between mosquito and mammalian cells (*11*), and adaptation of dengue virus to mosquito cells resulted in loss of the equivalent glycosylation motif (*12*), which suggests that the presence of carbohydrate on the E protein may be of lesser importance during virus replication in mosquito cells.

Recent data from the Mexican Department of Health indicate that no human cases of encephalitis attributable to local transmission of WNV have occurred during 2004 and, although many WNV-seropositive horses have been identified, few cases of overt clinical disease have been reported (http://www.cenave.gob.mx/von; accessed 26 Aug, 2004). The epidemiology of WNV disease in Mexico is likely to be complicated by preexisting immunity to other flaviviruses, but the emergence of an attenuated WNV strain would be important. WNV nucleotide sequences obtained from infected horses in Mexican states closer to the U.S. border suggest that they are closely related to recent isolates from Texas that do not encode mutations at the E glycosylation motif (*13*; J. Estrada-Franco, et al., unpub. data).We are unaware of any other WNV isolates from Tabasco or other southern regions of Mexico. Obtaining additional isolates from southern Mexico is important to determine if a nonglycosylated WNV population is emerging and to ascertain what impact this may have on the prevalence of severe WNV disease in Mexico.
